# Acute Respiratory Distress Syndrome Secondary to Enterovirus-Human-Rhinovirus Infection in an Adult

**DOI:** 10.7759/cureus.26475

**Published:** 2022-06-30

**Authors:** Khizar Hamid, Mohammad Ali, Joe Devasahayam

**Affiliations:** 1 Internal Medicine, University of South Dakota Sanford School of Medicine, Sioux Falls, USA; 2 Pulmonary Critical Care, Avera McKennan Hospital and University Health Center, Sioux Falls, USA

**Keywords:** acute respiratory distress syndrome (ards), covid 19, community aquired pneumonia, viral infection, medical intensive care unit (micu)

## Abstract

Enterovirus-human-rhinovirus (EV-HRV) are small RNA viruses that are airborne and can spread by direct contact or fomites and usually cause the common cold, asthma and chronic obstructive pulmonary disease exacerbation. EV-HRV-associated acute respiratory distress syndrome (ARDS) is common in children but is a rare cause of ARDS in adults. ARDS is defined according to the Berlin criteria and can be mild, moderate or severe depending on the PaO_^2^_ to FiO_2_ ratio. We report a case of a 70-year-old female with cardiac comorbidities, emphysema, second-hand smoking of 25 years, on methotrexate for rheumatoid arthritis presenting with ARDS secondary to EV-HRV infection. Despite initial treatment with appropriate antibiotics, steroids, low tidal volume mechanical ventilation, rescue maneuvers such as ventilation in prone positioning, paralyses, and inhaled nitric oxide, she passed away. EV-HRV causes upper respiratory tract infections but causes cytokine releases such as IL-1, IL-6, and IL-8 in the lower respiratory tract and in the blood which can cause ARDS. Very few cases of EV-HRV ARDS in immunocompetent adults are reported in the literature. Female sex is also associated with EV-HRV ARDS. No antiviral therapy exists for patients critically ill with EV-HRV; however, one case of successful treatment with high-dose intravenous vitamin C (HIVC) is reported in the literature. EV-HRV is one of the most common viruses identified in patients admitted with viral pneumonia in the intensive care unit. It should not be forgotten as a cause of ARDS.

## Introduction

Enterovirus-human-rhinovirus (EV-HRV) belongs to the Picornavirus family. These are small, nonenveloped, icosahedral viruses with positive-strand RNA genomes. Picornaviruses, which infect humans and cause high morbidity, belong to the *Enterovirus* genus [1]. These can be airborne and can spread via direct contact or contact with fomites [2]. EV-HRV are responsible for the common cold and are the usual culprits for asthma and chronic obstructive pulmonary disease exacerbation [3]. They mainly cause infections in the pediatric population but in adults the disease course is variable [4]. EV-HRV is a rare cause of acute respiratory distress syndrome (ARDS), with EV-HRV ARDS being more commonly seen in immunocompromised and elderly patients [4,5].

## Case presentation

**Figure 1:**
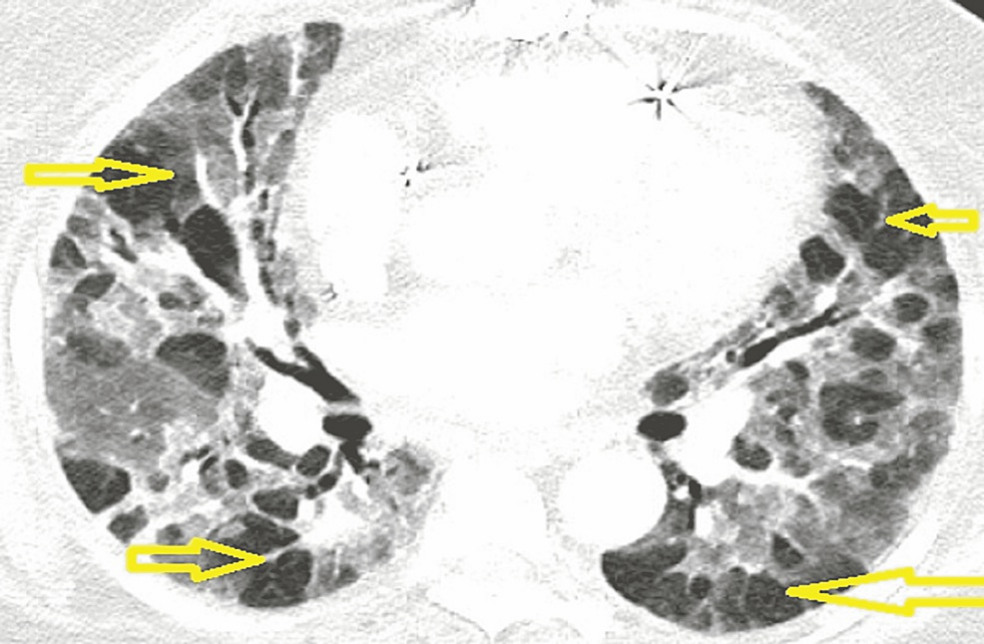
CTA PE showing severe emphysematous changes (yellow arrows) and extensive consolidation seen bilaterally. The patient was a second-hand smoker for 25 years. Her partner stopped smoking 15 years before the presentation. CTA PE - computed tomographic angiogram pulmonary embolism

A 70-year-old Caucasian female unvaccinated for COVID-19 was admitted to the hospital with complaints of shortness of breath for eight days. Her past medical history included chronic atrial fibrillation on sotalol with a pacemaker in situ, coronary artery disease treated with coronary artery bypass grafting, rheumatoid arthritis on methotrexate, hypertension, hyperlipidemia, hypothyroidism, obstructive sleep apnea on continuous positive airway pressure therapy, chronic kidney disease stage 3b and history of gastrointestinal bleeding (GIB). Before a scheduled endoscopy for GIB evaluation, she was found to have an oxygen saturation of 65% prompting her admission. She was noted to be morbidly obese with a BMI of 42 kg/m^2^ requiring 3L of oxygen via nasal cannula (NC) to maintain an oxygen saturation of 95%. Lab work was unremarkable. She was a nonsmoker but her husband was a smoker for 25 years quitting 15 years ago. A computed tomographic angiogram (CTA) of the chest was done which excluded pulmonary embolism (PE) but revealed severe emphysema and multifocal bilateral atypical pneumonia (PNA) (Figure 1). Testing for COVID-19, MRSA PCR, urine Legionella, and urine streptococcal antigen returned negative. A comprehensive respiratory panel was obtained from the nasopharynx returning positive for entero-rhinovirus. An echocardiogram was done which revealed an ejection fraction of 65%-70%. Empiric treatment with antibiotics was initiated but her oxygen requirements continued to rise eventually requiring constant bilevel positive airway pressure support. Fungal serologies, ANA, anti-myeloperoxidase antibody, and serine protease 3 antibody testing also returned negative. She completed five days of appropriate antibiotics and was started on 40 mg methylprednisolone twice a day however her oxygen requirements increased requiring intubation and mechanical ventilation. A bronchoscopy was performed which was unremarkable and repeat COVID-19 testing, fungal testing, and cultures from the bronchoalveolar lavage (BAL) resulted in negative. Her steroids were increased to 1 g methylprednisolone for three days which was tapered to 40 mg twice a day with little improvement. Dapsone 100 mg per day was initiated for *Pneumocystis jirovecii* prophylaxis. Hypersensitivity pneumonitis testing from the BAL returned positive for *Aureobasidium pullulans*. Despite the use of rescue maneuvers such as frequent prone position ventilation and inhaled nitric oxide (iNO), her PaO_2_/FiO_2_ ratio (P/F) remained persistently below 150 mmHg. Aggressive diuresis and paralysis with cisatracurium for 48 hours did not decrease her FiO_2_ requirements. She was on the ventilator for 15 days and had been in the hospital for 30 days. Due to her continued decline and lack of improvement, the family elected for comfort cares, and the patient was extubated and passed away.

## Discussion

Berlin criteria can be used to define ARDS according to P/F. It can be categorized as mild (P/F >200 to ≤300 mmHg), moderate (P/F > 100 to ≤200 mmHg), or severe (P/F ≤100 mmHg) [6]. Seasonal viruses are identified in 22-36% of community-acquired PNA patients requiring intensive care unit (ICU) admissions with Influenza and EV-HRV being most commonly detected [5]. EV-HRV commonly causes upper respiratory tract infections in children and adults; however, it also causes cytokine release such as IL-1, IL-6, and IL-8 in the lower respiratory tract and blood which can cause ARDS and viral PNA [4,5]. ARDS due to EV-HRV infection is common in the pediatric population, especially in patients with asthma; however, it is a rare cause of ARDS in adults [4]. Interestingly, most EV-HRV ARDS are documented in females [2-4]. Severe EV-HRV-associated PNA is more likely to be seen in immunocompromised individuals [7]. Very few cases of EV-HRV-associated ARDS in immunocompetent hosts have been reported in the literature [2]. Our patient's history of treatment with methotrexate and its adverse pulmonary effects and immunosuppression could have predisposed her to severe EV-HRV infection [8]. Her emphysematous changes noted on CTA could have also predisposed her to more severe disease. 

There are no specific treatment approaches for EV-HRV-associated ARDS. The usual care of ARDS including low tidal volume ventilation of 6 mL/kg should be employed [9]. Prone position ventilation has also been shown to improve severe ARDS mortality [10]. Our patient had temporary improvements in FiO_2_ with prone position ventilation; however, upon supination, her FiO_2_ requirements would gradually increase to previous levels. Inhaled nitric oxide (iNO) was also utilized with transient improvement in oxygenation. iNO has been shown to improve oxygenation levels temporarily but it has not shown any mortality benefit and can cause renal impairment [11]. It can be utilized as rescue therapy to improve oxygenation [12]. Dexamethasone (6 mg) has been shown to improve mortality in COVID-19 infection of patients requiring oxygen or mechanical ventilation [13]. Another trial revealed that 20 mg of dexamethasone for five days followed by 10 mg of dexamethasone for five days improves the duration of mechanical ventilation and overall mortality in moderate to severe ARDS [14]. These benefits could also possibly apply to ARDS due to EV-HRV. Currently, there is no antiviral therapy for critically ill adults with EV-HRV PNA [5]. Fowler III et al. reported successfully treating EV-HRV ARDS with high-dose intravenous vitamin C (HIVC) [15]. However, more research needs to be done to identify vitamin C as a therapeutic option. Clinical trials of HIVC for severe COVID-19 infection are underway, which might shed some insight into the treatment of other severe pulmonary viral infections with HIVC [16]. The capsid-binding anti-HRV agent pleconaril reduced the duration of uncomplicated HRV infection by one day but was not approved due to concerning drug-drug interactions. Intranasal recombinant interferon alfa-2b is effective for postexposure prophylaxis but not for established infection. More research needs to be done for antiviral treatments for patients critically ill with EV-HRV [5].

## Conclusions

EV-HRV, which commonly causes upper respiratory tract infections in the pediatric population, can occasionally cause severe ARDS in adults. It usually causes this in immunocompromised patients but can also cause it in immunocompetent individuals. Limited data show female sex is associated with EV-HRV ARDS. The treatment approach is similar to ARDS from other causes, such as low tidal volume ventilation, prone position ventilation, and steroids. No antiviral therapy exists for critically ill patients infected with EV-HRV. One case of successful treatment with HIVC is reported in the literature. More research needs to be done to identify antiviral treatments for critically ill patients with EV-HRV ARDS. ARDS due to other viruses should continue to be on the differential during the COVID-19 pandemic.
